# Untangling obese asthma: Design of proof-of-concept study of semaglutide in poorly controlled asthma

**DOI:** 10.1016/j.jacig.2025.100627

**Published:** 2025-12-17

**Authors:** Alessandra Tomasello, Leonard B. Bacharier, Patrice M. Becker, Caeden Dempsey, Pingsheng Wu, R. Stokes Peebles, Kevin Niswender, William D. Dupont, Gordon Bernard, Katherine N. Cahill

**Affiliations:** a Division of Allergy, Pulmonary and Critical Care Medicine, Department of Medicine, Vanderbilt University Medical Center, Nashville, Tenn; b Division of Endocrinology, Diabetes, and Metabolism, Department of Medicine, Vanderbilt University Medical Center, Nashville, Tenn; c Department of Biostatistics, Vanderbilt University Medical Center, Nashville, Tenn; d Division of Allergy, Immunology and Pulmonary Medicine, Department of Pediatrics, Monroe Carell Jr Children’s Hospital at Vanderbilt, Nashville, Tenn; e Division of Allergy, Immunology, and Transplantation, National Institute of Allergy and Infectious Diseases, Rockville, Md

**Keywords:** Asthma, adiposity, obesity, randomized-clinical trial, metabolic endotype

## Abstract

**Background:**

The intersection of obesity and asthma represents a complex clinical challenge characterized by increased symptom burden, reduced treatment efficacy, and multifactorial pathophysiology. Obesity-associated asthma is a heterogeneous condition shaped by underlying metabolic dysfunctions such as insulin resistance and altered inflammatory processes.

**Objectives:**

Current research often oversimplifies the relationship between obesity and asthma by relying primarily on body mass index as a measure, thereby overlooking key metabolic factors that may influence disease severity and treatment response. There is a critical need for clinical trials that account for this metabolic complexity, so we designed a proof-of-concept study with this in mind.

**Methods:**

Using the GLP-1R Agonists in the Treatment of Adult, Symptomatic, Obese Asthma (GATA-3) trial (ClinicalTrials.gov NCT05254314) as a conceptual framework, we propose an evolved model for future asthma research. While not a direct report of GATA-3 findings, it emphasizes the integration of comprehensive metabolic profiling—including insulin sensitivity and body composition—alongside traditional inflammatory and respiratory metrics in randomized controlled asthma trials.

**Results:**

The GATA-3 study design serves as an example of the first placebo-controlled trial to evaluate the glucagon-like peptide 1 receptor pathway’s role in asthma management independent of weight loss. The trial underscores essential design elements such as accurate asthma diagnosis, recognition of endotype heterogeneity, and implementation of outcome measures tailored to this phenotype.

**Conclusion:**

Advancing our understanding of obesity-associated asthma requires moving beyond body mass index–focused models to fully consider the metabolic complexity of the disease. Integrating detailed metabolic assessments into research and clinical practice will be vital for identifying responsive subpopulations, optimizing treatment strategies, and ultimately improving patient outcomes.

Asthma is a global health concern, affecting over 262 million people.^1^ At the same time, obesity has become a major public health challenge, contributing significantly to chronic diseases. The obesity epidemic is projected to affect asthma rates, with obesity expected to rise from 14% of the global population in 2020 to 25% by 2035, affecting around 1.9 billion people.^2^ Over the past two decades, the prevalence of asthma has also increased, with growing evidence linking obesity to both the development and worsening of the condition.^3^^,^^4^

The relationship between obesity and asthma is complex, with a bidirectional interaction: activity limitations caused by poorly controlled asthma and metabolic adverse effects of asthma therapies may contribute to the development of obesity, while obesity itself may increase the risk of asthma onset or aggravate ongoing asthma. Specifically, individuals with inadequate asthma control or those affected by severe forms of the disease often experience reduced physical activity resulting from breathlessness or other asthma-related respiratory symptoms, further increasing the likelihood of weight gain.^4^ Additionally, certain therapies used for the management of asthma, such as corticosteroids, can induce metabolic changes that promote weight gain and abdominal fat accumulation. In both scenarios, individuals tend to experience poorer asthma control, reduced quality of life, and higher rates of exacerbations, making obesity-associated asthma a particularly challenging condition to treat.^5^ Whatever the cause, obesity increases asthma symptom burden and reduces the response to inhaled corticosteroids.^6^^,^^7^

The mechanisms underlying the relationship between obesity and asthma are multifaceted, with systemic inflammation and metabolic dysfunction proposed to be key contributors. Growing evidence of a metabolic underpinning to asthma has opened new avenues for research, urging a closer examination of how metabolic factors interact with the airway and immune pathways to influence asthma outcomes. Many clinical studies demonstrate weight loss or targeting glycemic control can improve asthma outcomes in obese individuals.^8^, ^9^, ^10^, ^11^, ^12^, ^13^, ^14^, ^15^, ^16^ These studies have relied on body mass index (BMI) as the primary indicator of obesity or cohorts with overt type 2 diabetes mellitus (T2DM), which may not fully capture the complexities of metabolic dysfunction or its effect on asthma. The underlying mechanisms, such as insulin resistance and the complexity of inflammation, require more comprehensive evaluation that goes beyond traditional measures captured in conventional asthma studies. To better understand the nuanced relationship between metabolic dysfunction, obesity, and asthma, it is crucial to conduct randomized controlled trials that incorporate detailed metabolic assessments. Such trials can help identify responsive populations and novel predictive biomarkers to address the multifaceted nature of obesity-associated asthma and the unmet clinical needs of this population.

Targeting incretins, hormones that are secreted by the small intestine and brain stem in response to food intake to regulate weight homeostasis and glycemic control, has transformed the approach to the management of hyperglycemia and obesity. The GLP-1R Agonists in the Treatment of Adult, Symptomatic, Obese Asthma (GATA-3) study (ClinicalTrials.gov NCT05254314) is the first randomized, double-blind, placebo-controlled trial designed to evaluate the effect of the glucagon-like peptide 1 (GLP-1) incretin pathway in asthma. This ongoing randomized controlled trial aims to address our knowledge gap by examining the effects of metabolic interventions on asthma control and exploring the role of metabolic dysfunction independent of overt diabetes in shaping asthma outcomes. We will utilize the GATA-3 trial study design as a framework to discuss asthma clinical trial design focused on obesity-associated asthma populations.

Conventionally defined by BMI ≥ 30 kg/m^2^, obesity is more than an increase in body weight. Obesity contributes to metabolic dysfunction—including dyslipidemia, insulin resistance and hyperglycemia—and increases adipokine levels such as leptin, which activate proinflammatory pathways that further aggravate asthma.^17^ Mechanical changes in the chest wall and diaphragm may affect respiratory mechanics and airway hyperresponsiveness.^18^ Reduced pulmonary function in individuals with BMI ≥ 30 kg/m^2^ highlights the challenges faced determining the true cause of obesity’s impact on asthma.^19^ Importantly, when both BMI and insulin resistance are considered, insulin resistance appears to be a stronger predictor of forced expiratory volume in 1 second (FEV_1_) impairment than mechanical loading alone.^20^ Thus, disentangling the effect of excess fat mass from metabolic dysfunction on health is paramount to guide management decisions that can affect our patients whose BMI is above or below the arbitrary BMI = 30 kg/m^2^ cut point. Obesity also increases risk for comorbidities such as depression, obstructive sleep apnea, reflux, and cardiovascular diseases, all of which influence asthma outcomes.

Weight loss, even if modest (5-10%), has been shown to improve asthma control and lung function.^11^^,^^12^^,^^21^, ^22^, ^23^ Among interventions, dietary changes and bariatric surgery have both demonstrated clinical benefits, although effects on airway inflammation vary.^8^^,^^10^ Pharmacologic treatments targeting metabolic dysfunction may offer additional therapeutic options. Metformin therapy is associated with reduced asthma exacerbations and fewer emergency visits,^14^^,^^15^^,^^24^ highlighting the potential protective effects of metformin in asthma patients with comorbid T2DM. However, the impact of metformin on individuals without diabetes requires further research. Additionally, glucagon-like peptide 1 receptor agonists (GLP-1RAs), including semaglutide and liraglutide, known for their efficacy in controlling blood sugar and promoting weight loss, also show potential in modulating airway inflammation. Retrospective data suggest that GLP-1RAs may lower asthma exacerbation rates, independent of improvements in glycemic control and weight loss.^16^^,^^25^ A recent study from the UK Clinical Practice Research Datalink found that metformin significantly reduced asthma attacks, with even greater reductions when GLP-1RAs were added, underscoring their potential in managing asthma in patients with comorbid T2DM.^25^ Prospective studies of novel interventions targeting obesity and/or metabolism in carefully metabolically phenotyped patients with asthma provide an opportunity to move beyond BMI as a biomarker of disease and identify critical targets for therapeutic progress.

## Methods

### GATA-3 rationale

The GATA-3 clinical trial seeks to investigate the potential therapeutic effects of the GLP-1RA semaglutide in obesity-associated asthma, comparing its efficacy to that of placebo. By focusing on this dual condition, this study aims to provide critical insights into how obesity influences systemic and airway inflammation, metabolic dysfunction, and treatment response, ultimately contributing to the development of more targeted and effective interventions for affected individuals. Semaglutide is a GLP-1RA currently approved for the treatment of obesity and for overweight with a metabolic comorbidity (hypertension, T2DM, or dyslipidemia). Our preclinical data suggest that signaling through GLP-1R significantly inhibits allergic and viral airway inflammation in both lean and obese murine models. The GLP-1RAs liraglutide and semaglutide reduced allergen-challenge– and virus-induced lung IL-33 and subsequent pathways that contribute to airway cytokine generation, inflammation, mucus secretion, and hyperresponsiveness.^26^, ^27^, ^28^ Our cross-sectional clinical data in adults with both asthma and T2DM demonstrate that GLP-1RA decreases serum periostin levels; this biomarker is associated with airway inflammation and remodeling.^29^ In a new-user, active-comparator, retrospective cohort study, GLP-1RA therapy reduced the risk of asthma exacerbation compared to other diabetes-control medications.^16^ Asthma exacerbation risk reduction with GLP-1RA therapy in T2DM was further supported in a UK population-based cohort.^25^ The reduction in exacerbation risk was independent of weight loss or glycemic control in both T2DM cohorts. Together, these data support the hypothesis to be tested that GLP-1RAs improve asthma control and reduce airway inflammation as a result of direct effects on the respiratory tract in obesity-associated asthma.

### GATA-3 study design

**Fig 1:**
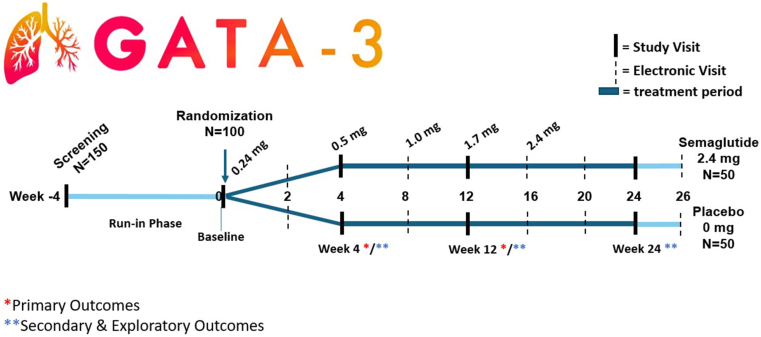
GATA-3 study schema illustrating sequence of scheduled visits throughout study period. In-person study visits include spirometry, questionnaire administration, and biological sample collection. Electronic visits include remote assessment of asthma control (ACQ-6), monitoring of treatment compliance, and reporting of adverse events. schema also outlines stepwise escalation of investigational drug in accordance with US Food and Drug Administration guidelines. Time points are indicated relative to start of intervention (week 0). Table I lists primary and secondary outcomes.

The GATA-3 trial is a phase 2 proof-of-concept study designed to test the central hypothesis that semaglutide, a GLP-1RA, can improve asthma control and reduce airway inflammation in individuals with obesity-associated asthma through direct effects on the respiratory tract. The GATA-3 study is a randomized, double-blind, placebo-controlled trial evaluating the effects of semaglutide 0.24-2.4 mg in the context of asthma and will provide novel information on the effect of GLP-1RA on asthma outcomes without comorbid diabetes. After a 4-week run-in period (to document poorly controlled asthma despite adherence to medium-dose inhaled corticosteroids or more therapy), study participants will be randomized to semaglutide 2.4 mg via current US Food and Drug Administration label dose escalation or matched placebo administered subcutaneously once a week for 24 weeks, followed by a 2-week final monitoring period (Fig 1). This approach will assess whether a signaling pathway known to regulate metabolic homeostasis also plays a role in controlling airway inflammation in patients with asthma and comorbid obesity. Through this study, we aim to advance our understanding of the therapeutic potential of GLP-1RAs in asthma, specifically in the setting of obesity, by evaluating their direct effect on the airway.

## Results

### Study objectives and end points

For this US National Institutes of Health–funded proof-of-concept clinical trial, both clinical and mechanistic end points have been selected (Table I). Notably, the phase 3 trial of semaglutide 2.4 mg weekly in adults with overweight or obesity demonstrated a mean body weight change that does not exceed −2% by week 4 and −5% over placebo by week 12.^30^ Therefore, we selected early time points for our outcome measures when the effect of weight loss would be minimal. At these time points, doses of semaglutide are low (0.24-1.0 mg, Fig 1) and have not yet reached the dose approved for the management of obesity (2.4 mg). The primary clinical objective is to assess the efficacy of once-weekly semaglutide in improving asthma control in individuals with symptomatic, persistent asthma and obesity. It is the difference between the treatment and placebo groups in the change from baseline in the Asthma Control Questionnaire (ACQ)-7 score at week 12. ACQ-7, a widely recognized tool for evaluating asthma control over a 7-day period, provides a combined assessment of current clinical status and future risk in FEV_1_, the fundamental goals of any asthma therapy. A reduction in ACQ-7 score compared to placebo at week 12 would support the hypothesis that semaglutide improves asthma beyond its role in weight reduction and at doses lower than those approved for weight loss.

**Table I:** GATA-3 clinical and mechanistic outcomes

Outcome	Outcome measure	Measure description
Primary	Efficacy of semaglutide on ACQ-7 score in subjects with symptomatic, persistent asthma and obesity at week 12	Primary clinical outcome is difference between treatment and placebo groups in change from baseline in ACQ-7 score to week 12
	Effect of semaglutide once weekly on serum periostin in subjects with symptomatic, persistent asthma and obesity at week 4	Primary mechanistic outcome is difference between treatment and placebo groups in change from baseline in serum periostin at week 4
Secondary	Effect of semaglutide on weight loss to week 24	Change from baseline in weight to week 24
	Efficacy of semaglutide once weekly on ACQ-6 score in subjects with symptomatic, persistent asthma and obesity to week 12	Change from baseline in ACQ-6 score to week 12
	Maximal dose of semaglutide tolerated in persistent asthma with obesity to week 24	Maximum tolerated dose of investigational product at week 24
	Change in exhaled nitric oxide from semaglutide to week 12	Change from baseline in exhaled nitric oxide at weeks 4 and 12
	Change in serum periostin from semaglutide to week 12	Change from baseline in serum periostin at week 12

The primary mechanistic objective is to evaluate the effect of once-weekly semaglutide on airway inflammation, independent of weight loss. The primary mechanistic outcome is the difference between the treatment and placebo groups in the change from baseline in serum periostin levels at week 4. Periostin is a biomarker associated with airway inflammation and remodeling, key components of asthma pathophysiology.^31^ Previous studies have suggested that GLP-1RA therapy is associated with lower periostin levels in a cross-sectional cohort with asthma and T2DM, which may reflect a reduction in airway inflammation and remodeling processes.^29^ Together, these outcomes address both the effectiveness of semaglutide in improving asthma control and the mechanisms through which it might reduce inflammation in the airway. Achieving these end points would not only show clinical benefit for asthma management in obese individuals but also reveal a potential new role for GLP-1RA in asthma treatment, independent of their effect on weight.

The study includes a set of secondary (Table I) and exploratory outcomes measured after longer exposure and the maximal 2.4 mg dose of semaglutide, obtained at week 16 and sustained at week 24, to determine whether any direct effects of semaglutide on airway inflammation are additive or synergistic to the effects of weight loss. Clinical exploratory outcomes include changes from baseline in Asthma Quality of Life Questionnaire scores and FEV_1_, and the rate of asthma exacerbations and the time to first exacerbation over the 24-week study period. Mechanistic exploratory outcomes include changes in airway inflammation and changes in metabolic biomarkers—including fasting insulin, lipid profile, hemoglobin A1c, body weight, waist-to-hip ratio, and body composition assessed via dual-energy X-ray absorptiometry scan.

### Study population

When designing a randomized controlled trial to evaluate treatments for obesity-associated asthma, it is essential to consider the unique characteristics and challenges of the asthma population with obesity. Although obese individuals with asthma exhibit similar levels of eosinophilic airway inflammation to their lean counterparts, they often face more complex airway inflammation, heightened respiratory symptom burden, poorer quality of life, and reduced lung function.^32^ These complexities emphasize the importance of tailoring clinical trial protocols to reflect their distinct needs. Moreover, the trial design should incorporate the growing body of evidence that supports personalized asthma management strategies for obese patients. They represent a high-risk group that is more susceptible to exacerbations and poorer treatment outcomes. As such, it is important to include not only patients with severe forms of the disease but also those with less severe forms, as both groups may benefit from tailored treatment approaches.^33^ It is crucial to avoid excluding participants solely on the basis of obesity-related comorbidities, such as sleep apnea or cardiovascular disease, which are prevalent in this group. The GATA-3 study includes adults with obesity (BMI ≥ 30 kg/m^2^ alone, or BMI ≥ 27 kg/m^2^ with obesity-related comorbidity) and persistent asthma requiring medium-dose or higher inhaled corticosteroids. To be eligible, participants must also have symptomatic asthma (defined by ACQ-6 score ≥ 1.5) and a stable asthma regimen for at least 8 weeks. However, individuals with diabetes, frequent short-acting bronchodilator receipt (>8 puffs/inhalations on most days in the previous week), other chronic respiratory diseases, recent weight loss treatments, or those receiving systemic glucocorticoids or monoclonal antibodies for asthma are excluded. Liberal allowances for baseline percentage predicted FEV_1_, renal impairment (estimated glomerular filtration rate ≥ 30 mL/min/1.73 m^2^), stable chronic metformin therapy, remote bariatric surgery, metabolic comorbidities, and the absence of conventional asthma biomarker cutoffs were included to maximize the target population in line with the reality of obesity-associated asthma in our clinics. A detailed breakdown of inclusion and exclusion criteria is provided in Table II. By carefully considering the distinct pathophysiologic and clinical characteristics of the asthma population with obesity, the trial protocol can more accurately reflect real-world conditions and enhance the relevance and applicability of the trial’s findings.

**Table II:** GATA-3 inclusion and exclusion criteria

Inclusion criteria 1. Subject must be able to understand and provide informed consent. 2. Male and female subjects aged 18 or older. 3. Obesity defined as BMI ≥ 30 kg/m^2^, or as BMI ≥ 27 kg/m^2^ in the setting of ≥1 weight-related comorbidity: a Clinically documented hypertension (>130 mmHg systolic or >85 mmHg diastolic or treatment) in prior year or during run-in. b Clinically documented dyslipidemia (triglycerides > 150 mg/dL, high-density lipoprotein < 40 mg/dL in male participants or < 50 mg/dL in female participants, low-density lipoprotein ≥130 mg/dL or treatment) in prior year or during run-in. c Current obstructive sleep apnea treatment. d Documented prediabetes defined by HbA1c > 5.7 and HbA1c < 6.5 in prior year or during run-in. e Clinically documented cardiovascular disease. 4. History of physician-diagnosed asthma. 5. Persistent Asthma as determined by the requirement of at least medium-dose daily inhaled corticosteroid or more. 6. Symptomatic asthma with an ACQ-6 score ≥ 1.5 at enrollment and at the time of randomization. 7. Patient report of stable asthma controller regimen for the prior 8 weeks. 8. Evidence of bronchodilator responsiveness (≥12% and at least 200 mL increase in FEV_1_) or airway hyperresponsiveness with a methacholine provocative concentration (PC)_20_ ≤ 16 mg/mL or provocative dose (PD)_20_ ≤ 400 μg in prior year. 9. Female subjects of childbearing potential must have a negative pregnancy test on study entry. 10. Female subjects of childbearing potential must agree to use a highly effective birth control method (eg, hormonal, surgical, or abstinence) for the duration of the study.
Exclusion criteria
*At enrollment:* 1. Inability or unwillingness of subject to give written informed consent or comply with study protocol. 2. Diagnosis of type 1 or type 2 diabetes mellitus or HbA1c ≥ 6.5% on screening laboratory studies. 3. More than 8 puffs/inhalations of short-acting bronchodilators is needed most days in previous week (ie, answer to question 6 on ACQ-6 = 4, 5, or 6). 4. Oxygen saturation < 94% on room air. 5. Patient-reported tobacco, e-cigarette, or inhaled marijuana use in last 12 months, or >10 pack years of use.∗ 6. Pregnancy by urine testing, current lactation, or plans to become pregnant during study period. 7. Pharmaceutical weight loss treatment for >7 days in prior 90 days at enrollment. 8. Previous surgical weight loss treatment. Can still be enrolled if surgery was >5 years ago and evidence exists of stable or increasing weight in prior 3-12 months. 9. Personal history of pancreatitis. 10. Personal or family history of medullary thyroid cancer or multiple endocrine neoplasia syndrome type 2. 11. Personal history of gallstone disease without previous cholecystectomy. 12. Personal history of gastroparesis. 13. Personal history of hypersensitivity to semaglutide. 14. Personal history of hypersensitivity to local amide type anesthetics (eg, lidocaine). 15. Receipt of antidiabetic agent, other than metformin, including GLP-1RA, in previous 90 days. Metformin is allowed, provided dose has been stable in 90 days before screening and will remain stable for duration of trial. 16. Receipt of systemic glucocorticoids in past 28 days. 17. Receipt of monoclonal antibody for treatment of asthma in past 120 days. 18. Myocardial infarction, unstable angina, stroke, or heart failure (New York Heart Association class II) within 1 year by history. 19. Patient report and confirmed by review of historical diagnostic testing by study physician of other physician-diagnosed chronic respiratory diseases: chronic obstructive pulmonary disease, cystic fibrosis, pulmonary hypertension, interstitial lung disease, sarcoidosis, bronchiectasis. 20. History of physician-diagnosed immune deficiency. 21. History of physician-diagnosed malignancy (other than excised nonmelanoma skin cancer) in past 5 years. 22. Current uncontrolled hypertension (>150 mmHg systolic, >90 mmHg diastolic) or untreated hyperthyroidism. 23. Current diagnosed or self-reported drug or alcohol abuse that, in investigator’s opinion, would interfere with subject’s ability to comply with study requirements. 24. Receipt of investigational drugs within 20 weeks of participation, other than vaccines and/or treatments for SARS-CoV-2 authorized for emergency use. 25. Past or current medical problems or findings from physical examination or laboratory testing that are not listed above, which, in investigator’s opinion, may pose additional risks from participation in study, may interfere with subject’s ability to comply with study requirements, or may affect quality or interpretation of data obtained from study.
*At randomization:* 26. Screening creatinine elevation with estimated glomerular filtration rate < 30 mL/min/1.73 m^2^ collected at visit 1. 27. Compliance to baseline asthma inhaler therapy of <80% during run-in, at time of randomization.

*DLCO,* Diffusing capacity of lung for carbon monoxide; *FVC,* forced vital capacity; *HbA1c,* hemoglobin A1c; *PC,* provocative concentration; *PD,* provocative dose; *SARS-CoV-2,* severe acute respiratory syndrome coronavirus 2.

∗ Can still be enrolled if ≥40 years old, smoked <20 pack years, none within 12 months, and demonstrate postbronchodilator FEV_1_/FVC ratio of >0.7 or DLCO *z* score of −1.645 or greater (or equivalent ≥ 75% of predicted) documented in prior 12 months.

## Discussion

### Specific considerations for obesity-associated asthma target populations

#### Confirming asthma diagnosis

The diagnosis of asthma in patients with obesity is often complicated by symptom overlap between obesity and asthma. Both conditions share clinical features, including shortness of breath, dyspnea with exertion, noisy breathing, and fatigue, which can lead to diagnostic ambiguity. Obesity-related physiologic changes, such as reduced lung volume and increased airway resistance, can mimic asthma symptoms, further complicating differentiation. It is well established that obesity alters respiratory system mechanics, affecting lung volumes and chest wall expansion as well as increasing the work of breathing. Excess trunk fat restricts chest wall expansion, elevates intra-abdominal and pleural pressures, and reduces expiratory reserve volume and functional residual capacity.^34^ Zhang et al^19^ found that obesity was associated with reduced pulmonary function, particularly lower FEV_1_ and forced vital capacity, with a U-shaped relationship between BMI and lung function, also observed with other body measurements such as waist circumference, body fat percentage, and body roundness index.

Given the potential for symptom overlap and impaired lung function in obese individuals, it is critical to confirm a diagnosis of asthma in this target population through objective pulmonary function tests. In the GATA-3 study, to ensure that participants have a definitive diagnosis of asthma, strict inclusion criteria are applied. Participants must demonstrate positive response to bronchodilator test defined as a ≥12% and ≥200 mL increase in FEV_1_. If the bronchodilator test is negative, participants must show a positive methacholine challenge (provocative concentration [PC]_20_ ≤ 16 mg/mL or provocative dose [PD]_20_ ≤ 400 μg), indicative of airway hyperresponsiveness. This approach ensures that only individuals with confirmed asthma with a measure of airway lability, as opposed to obesity-related respiratory symptoms alone, are included in the study cohort. The methacholine challenge test is of particular importance in the context of obesity-associated asthma. Traditional spirometry may fail to detect airway reactivity as a result of the confounding effects of excess adiposity, which can lead to altered airway dynamics.^18^ This underscores the challenges of diagnosing asthma in obese patients, where bronchodilator responsiveness may be diminished or absent as a result of mechanical and inflammatory effects of excess adiposity on the airways. By incorporating methacholine challenge, we can more reliably identify asthma in this population and distinguish it from other respiratory issues commonly associated with obesity.

#### Inflammatory endotyping

Asthma is not a single uniform condition but comprises various endotypes, which are influenced by comorbidities. Metabolic dysfunction linked to obesity can affect both type 2 (T2)-high and T2-low asthma endotypes, further complicating disease pathophysiology. Late-stage asthma clinical trials have relied on traditional inflammation assessment tools, such as fractional exhaled nitric oxide (Feno), total IgE levels, and peripheral blood eosinophils, to stratify T2-high from T2-low endotypes. Secondary analysis of asthma studies suggests the conventional cutoffs selected for phase 3 biologic studies are not applicable in the context of obesity.^35^ Although Feno is commonly used in clinical practice and asthma clinical trials to measure eosinophilic airway inflammation, its correlation with inflammation is weakened in individuals with obesity.^35^ This limitation stems from the effect of obesity on metabolic function, which alters the inflammatory profile of asthma and complicates its management. Research has suggested that an imbalance in metabolism, characterized by lower levels of l-arginine and higher levels of asymmetric dimethylarginine, may impair nitric oxide (NO) synthesis by uncoupling NO synthase, resulting in the production of reactive oxygen species instead of NO.^36^ This shift reduces NO availability, contributing to oxidative stress, impaired airway dilation, and diminished lung function, which in turn leads to poorer asthma control.^32^

To address these challenges, the GATA-3 study will utilize a range of alternative mechanistic assays designed to assess airway inflammation (Table III). These will complement Feno and blood eosinophil measurements, allowing for a more comprehensive evaluation of inflammation. Induced sputum analysis and upper airway sampling will be incorporated to better capture the inflammatory response in the airways, particularly in the context of obesity-associated asthma.

**Table III:** Mechanistic assays designed to assess airway inflammation in GATA-3 study

Hypothesis	Measure	Time point	Importance
GLP-1RA reduce T2 and non-T2 airway inflammation in obesity-associated asthma before weight loss from GLP-1RA is observed	Periostin (serum)	Weeks 4 and 12	• Confirms T2 and non-T2 anti-inflammatory effect in airway independent of weight loss. • Supports use in nonobese patient population.
	Sputum and upper airway sampling	Weeks 4 and 12	
Weight loss will synergize with effect of GLP-1RA to further reduce T2 and non-T2 airway inflammation	Periostin (serum)	Week 24	• Confirms T2 and non-T2 anti-inflammatory effect in airway after weight loss. • Supports prescribing maximal doses of GLP-1RA for treatment of obesity-associated asthma.
	Sputum and upper airway sampling	Week 24	

#### Adiposity and metabolic assessments

Given the growing knowledge about how insulin resistance and hyperglycemia may alter asthma and treatment response, it is critical that metabolic assessments are included in clinical and research evaluations. Because obesity-related metabolic dysfunction could modify the response to asthma treatments, the GATA-3 study will systematically monitor metabolic parameters, including lipid profile (triglycerides, total cholesterol, high-density lipoprotein, and low-density lipoprotein), A1c, fasting insulin, and fasting glucose at baseline and throughout treatment. These measures will help measure insulin resistance and metabolic dysfunction, assessing their effect on asthma outcomes. This metabolic profiling will provide valuable insights into how metabolic health influences therapeutic efficacy, leading to more tailored interventions for patients with obesity-associated asthma. By integrating these advanced measurements, we aim to better define *metabolic asthma* and provide more refined metabolic endotyping, ultimately guiding personalized treatment strategies for this unaddressed subpopulation. Furthermore, traditional measures of obesity, such as weight and BMI, offer limited insight into body composition because they do not distinguish between fat mass and lean mass or their distribution. BMI remains a widely used tool for assessing obesity, but it fails to fully capture the complexities of body composition, thus potentially leading to an incomplete understanding of the relationship between obesity, asthma, and associated metabolic comorbidities.

To address this limitation, our study incorporates both routine anthropometric measurements (weight, height, and waist and hip circumference) and dual-energy X-ray absorptiometry for fat mass assessments. This comprehensive approach will not only distinguish between central and peripheral obesity but also assess overall body composition. Body composition will be evaluated at baseline and at 4, 12, and 24 weeks to track changes over time and examine their correlation with asthma severity, symptom control, and treatment response and provide additional insight into the impact of body composition change on our clinical and mechanistic outcomes. This evaluation will also provide critical insights into fat mass and lean mass, which are essential for understanding the diverse effects of obesity and the body’s response to interventions. Table IV provides an overview of relevant metabolic parameters for obesity-associated asthma.

**Table IV:** Overview of metabolic parameters for obesity-associated asthma

Characteristic	Relevance	Feasibility
BMI	Widely used to assess obesity but does not differentiate between fat mass and lean mass. It provides an overall measure of body weight relative to height.	Easy to measure and noninvasive. However, it lacks precision in differentiating between fat and muscle mass, making it less informative regarding the relationship between obesity and asthma.
Anthropometric measurements	Includes weight, height, waist circumference, and hip circumference. Helps assess obesity phenotype (central vs peripheral obesity) and provide insight into body composition.	Relatively simple and noninvasive. Commonly used in clinical settings, although less precise than advanced techniques like DEXA. Waist-to-hip ratio is a good indicator of central obesity.
Assessment of body composition via DEXA	Provides precise measurements of total body fat, lean mass, and regional fat depots (eg, visceral adipose tissue). Crucial for understanding the role of fat mass, especially visceral fat, in asthma.	Reference standard for body composition analysis. Requires specialized equipment and trained personnel, making it less accessible and more expensive. However, it provides highly accurate data on body composition and fat distribution
Hemoglobin A1c	Reflects long-term blood glucose control and is a key marker for assessing insulin resistance and metabolic health. Elevated A1c levels are associated with poor metabolic health and asthma severity.	Easy to measure with standard laboratory tests. Noninvasive and commonly used in clinical settings. Less effective for identifying short-term fluctuations in blood glucose levels, but useful for long-term monitoring
Fasting insulin and assessment of insulin resistance	Insulin resistance is linked to obesity and asthma severity. Insulin resistance is associated with worsening lung function.	Relatively easy to measure through blood tests but requires fasting.
Lipid panel	Dyslipidemia, often present in individuals with obesity and insulin resistance, is a key metabolic dysfunction that may exacerbate inflammation.	Commonly used in clinical practice. Relatively simple and accessible. Provides important information about lipid metabolism, which may influence asthma severity and response to treatment.

*DEXA,* Dual-energy X-ray absorptiometry.

### Study considerations and limitations

The GATA-3 study, as with all research investigations, is constrained by available resources. Although our findings will contribute to the growing body of evidence on the role of metabolic dysfunction in asthma pathophysiology, we must address several important limitations. The absence of a control arm examining weight loss through alternative interventions like diet and exercise limits our ability to directly compare the effectiveness of semaglutide with more traditional weight management strategies. Because lifestyle modifications are commonly used in asthma management, future trials incorporating such interventions would provide valuable insights into how semaglutide compares to these approaches in addressing metabolic dysfunction and asthma outcomes. Furthermore, our study does not include comprehensive lung function assessments, particularly measures of lung volume and oscillometry, which could offer a more detailed understanding of the effect on airway resistance and lung mechanics. These measurements would help clarify the role of metabolic dysfunction in asthma pathophysiology and intervention response. Similarly, the lack of bronchial tissue biopsy samples prevents us from evaluating the local effects on airway inflammation at the tissue level. Future studies incorporating tissue sampling are essential to understanding the direct effect of metabolic interventions on airway inflammation and tissue-level metabolic changes. Additionally, the absence of IgE measurement and skin prick testing limits our ability to characterize atopic status and explore potential interactions between allergic sensitization and metabolic dysfunction in asthma. The inclusion of these tools would enhance phenotypic characterization and may help identify subgroups more likely to respond to metabolic therapies. The inclusion of dietary assessments, activity monitoring, impulse oscillometry, chest imaging for air trapping and mucus plugging, endobronchial biopsies, repeated reactivity assessments, measures of systemic inflammation (C-reactive protein, erythrocyte sedimentation rate) would add value to future investigations of obesity-associated asthma.

### Conclusion

**Fig 2:**
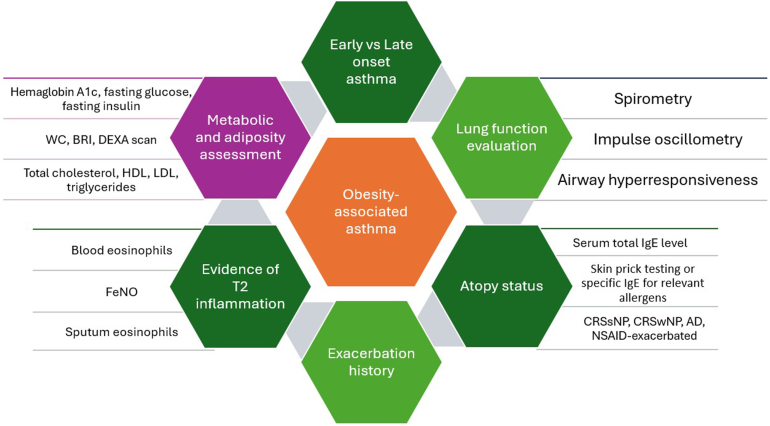
Conceptual model to guide future studies in redefining obesity-associated asthma phenotype. *BRI,* Body roundness index; *CRSwNP,* chronic rhinosinusitis with nasal polyps; *CRSwNP,* chronic rhinosinusitis without nasal polyps; *DEXA,* dual-energy X-ray absorptiometry; *NSAID,* nonsteroidal anti-inflammatory drug; *WC,* waist circumference.

Research studies and clinical trials on obesity-associated asthma should reconsider their design by moving beyond a sole focus on obesity status, typically defined by BMI ≥ 30 kg/m^2^. Instead, greater attention must be given to the complex features that characterize obesity, particularly the metabolic alterations associated with it. A redefined approach to phenotyping obesity-associated asthma is needed—one that incorporates comprehensive evaluations of both adiposity and metabolic function as well as classic phenotyping strategies in asthma (Fig 2). Additionally, most studies have included comorbid diabetes, limiting our application to patients without diabetes. Future research should distinguish between the comorbid metabolic alterations that often accompany obesity, such as diabetes, insulin resistance, and dyslipidemia. Our research represents a paradigm shift by exploring the role of the incretin signaling pathway in regulating both metabolic homeostasis and airway inflammation. In its focus on the therapeutic potential of GLP-1RA, this study lays the groundwork for future research that may transform the treatment of obesity-associated asthma. Investigating the direct effects of GLP-1RA on the airway and assessing the broader effect of antiobesity medications on asthma outcomes could revolutionize treatment strategies.

**Key messages**

• Obesity-associated asthma is a metabolically heterogeneous condition that requires research models moving beyond BMI to incorporate detailed assessments of insulin resistance, body composition, and inflammation.
• The GATA-3 trial framework illustrates how incorporating comprehensive metabolic and inflammatory profiling can improve the design and clinical relevance of asthma trials.
• GLP-1 receptor agonists represent a promising therapeutic avenue for obesity-associated asthma, with potential effects on metabolic regulation and airway inflammation.

## Disclosure statement

Supported by the National Institutes of Health (NIH; grants U01 AI155299 and UL1 TR000445). P.M.B.’s and C.D.’s coauthorship of this report does not necessarily represent the views of the National Institute of Allergy and Infectious Diseases, the NIH, or any other agency of the United States government.

Disclosure of potential conflict of interest: K. N. Cahill served on scientific advisory boards for AstraZeneca, Sanofi, Genentech, Regeneron, Novartis, GSK, and Eli Lilly; served as a consultant for Ribon Therapeutics, Third Harmonic Bio, Ramble Health, Apogee, and Verantos; reports royalties from UpToDate; and reports research support from Novo Nordisk. L. B. Bacharier is a member of the GINA Science Committee; reports grants from the NIH, National Institute of Allergy and Infectious Diseases, and National Heart, Lung, and Blood Institute; reports personal fees from GlaxoSmithKline, Genentech/Novartis, AstraZeneca, Avillion, WebMD/Medscape, Sanofi/Regeneron, Circassia, OM Pharma, Apogee, and Kinaset; for DSMB from AstraZeneca, DBV Technologies, Aravax, and Vertex; and royalties from Elsevier and UpToDate outside the current report. The rest of the authors declare that they have no relevant conflicts of interest.
